# Zymogen and activated protein C have similar structural architecture

**DOI:** 10.1074/jbc.RA120.014789

**Published:** 2020-08-27

**Authors:** Bosko M. Stojanovski, Leslie A. Pelc, Xiaobing Zuo, Enrico Di Cera

**Affiliations:** 1 Edward A. Doisy Department of Biochemistry and Molecular Biology, Saint Louis University School of Medicine, St. Louis, Missouri, USA; 2 X-Ray Science Division, Argonne National Laboratory, Lemont, Illinois, USA

**Keywords:** protein C, thrombin, serine protease, single molecule biophysics, protein conformation, ligand-binding protein, single-molecule biophysics

## Abstract

Activated protein C is a trypsin-like protease with anticoagulant and cytoprotective properties that is generated by thrombin from the zymogen precursor protein C in a reaction greatly accelerated by the cofactor thrombomodulin. The molecular details of this activation remain elusive due to the lack of structural information. We now fill this gap by providing information on the overall structural organization of these proteins using single molecule FRET and small angle X-ray scattering. Under physiological conditions, both zymogen and protease adopt a conformation with all domains vertically aligned along an axis 76 Å long and maximal particle size of 120 Å. This conformation is stabilized by binding of Ca^2+^ to the Gla domain and is affected minimally by interaction with thrombin. Hence, the zymogen protein C likely interacts with the thrombin-thrombomodulin complex through a rigid body association that produces a protease with essentially the same structural architecture. This scenario stands in contrast to an analogous reaction in the coagulation cascade where conversion of the zymogen prothrombin to the protease meizothrombin by the prothrombinase complex is linked to a large conformational transition of the entire protein. The presence of rigid epidermal growth factor domains in protein C as opposed to kringles in prothrombin likely accounts for the different conformational plasticity of the two zymogens. The new structural features reported here for protein C have general relevance to vitamin K-dependent clotting factors containing epidermal growth factor domains, such as factors VII, IX, and X.

Protein C is a glycoprotein with modular architecture similar to that of other vitamin K-dependent factors of the blood coagulation cascade, *i.e.* prothrombin, factors VII, IX, and X (1). Synthesis of protein C in the liver produces a zymogen form of 419 amino acids comprising the N-terminal Gla domain (residues 1-46), two epidermal growth factor (EGF)-like domains (residues 55-90 and 96-136), and a C-terminal trypsin-like domain (residues 170-419). Three linker regions connect the Gla domain to EGF1 (residues 47-54), the two EGF domains (residues 91-95) and EGF2 to the protease domain (residues 137-169). This last segment contains the activation peptide (residues 158-169) and the dipeptide sequence Lys^156^–Arg^157^ that is removed during synthesis to produce a two-chain zymogen where the light chain (residues 1-155) and heavy chain (residues 158-419) remain connected through a disulfide bond between Cys^141^ and Cys^277^ (Fig. 1*A*). It is in this two-chain form that 90% of protein C circulates in the plasma, with the rest being slightly modified but functionally equivalent (2).

**Figure 1.**
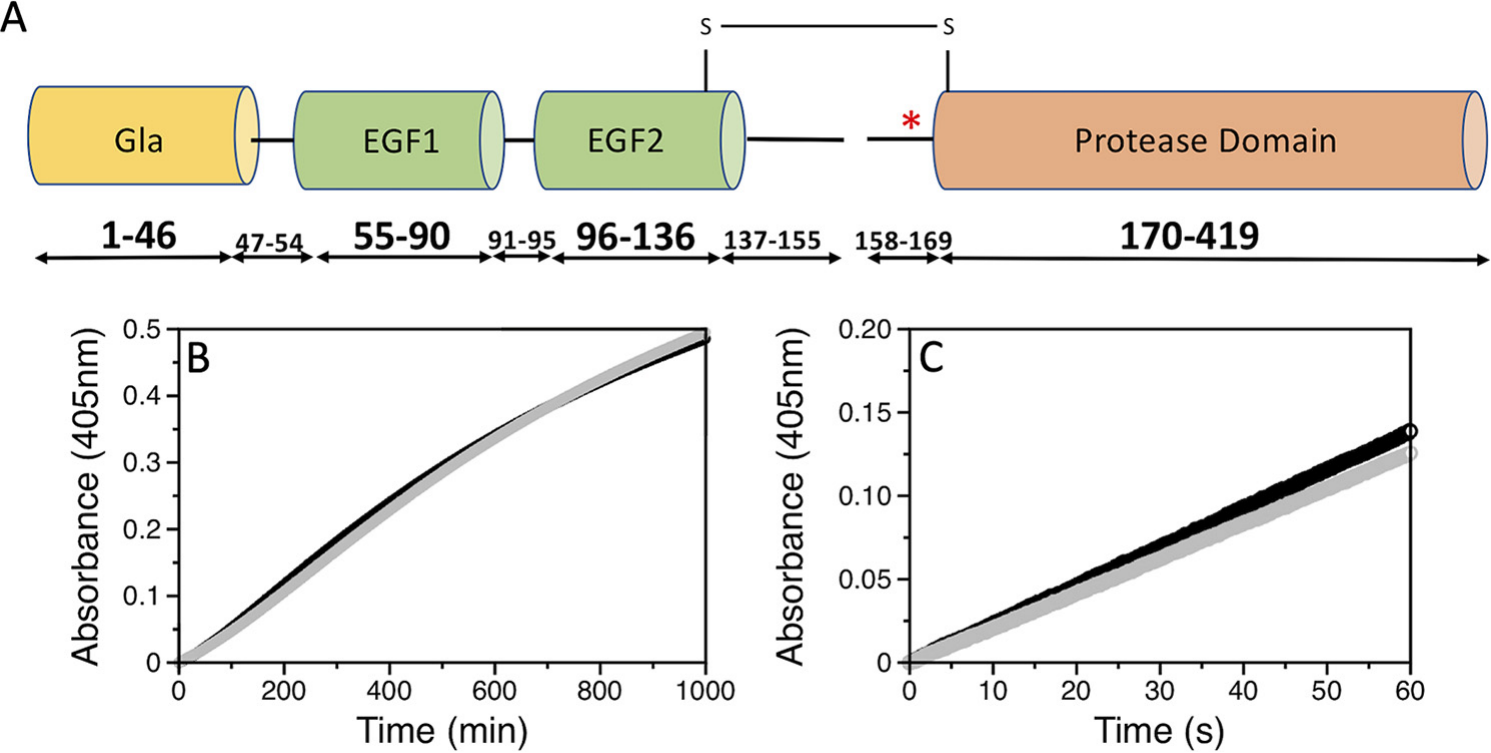
*A,* schematic representation of protein C comprising the Gla (residues 1-46), EGF1 (residues 55-90), EGF2 (residues 96-136), and protease domains (residues 170-419). Three linkers connect the different domains. A dipeptide consisting of residues Lys^156^ and Arg^157^ is removed from the third linker to produce a two-chain zymogen where the light chain (residues 1-155) and heavy chain (residues 158-419) remain connected through the Cys^141^–Cys^277^ disulfide bond. Cleavage by thrombin at Arg^169^ removes the activation peptide (*red asterisk*, residues 158-169) and produces APC. *B,* thrombin-catalyzed activation of the AF555/AF647 labeled (*black*) and unlabeled (*gray*) protein C S12C/R312C mutant. Activation was monitored by a progress curve using the APC-specific substrate H-D-Asp-Arg-Arg-*p*-nitroanilide at 405 nm under experimental conditions: 20 mm Tris, 145 mm NaCl, 5 mm EDTA, 0.1% PEG8000, pH 7.5, at 37 °C. *C,* hydrolysis of the chromogenic substrate S-2366 by the AF555/AF647-labeled (*black*) and unlabeled (*gray*) APC S12C/R312C mutant monitored at 405 nm under experimental conditions: 20 mm Tris, 145 mm NaCl, 5 mm CaCl_2_, 0.1% PEG 8000, pH 7.5, at 25 °C. Saturating amounts of the inhibitor hirudin (250 nm) were added to rule out contamination by thrombin activity.

Protein C is activated by thrombin upon cleavage at Arg^169^, resulting in removal of the entire activation peptide and folding of the active site and primary specificity pocket as in other members of the trypsin family (3). Activated protein C (APC) inactivates cofactors Va and VIIIa with the assistance of protein S, down-regulates the amplification and progression of the coagulation cascade, and maintains patency of the capillaries (4, 5). As an anti-inflammatory and cytoprotective agent, APC signals through PAR1 and PAR3 in ways that differ completely from thrombin's activation mechanism and reduces cellular damage following sepsis and ischemia/reperfusion of the brain, heart, lungs, and kidneys (6, 7).

Activation of protein C by thrombin is highly inefficient and requires the endothelial receptor thrombomodulin to enhance the rate >1,000-fold to a level compatible with physiological function (8–10). An additional improvement of the reaction rate is contributed by the endothelial protein C receptor (4). The molecular mechanism leading to protein C activation remains poorly understood because structural information is limited to Gla-domainless APC (11), with no available data on protein C free or bound to the thrombin-thrombomodulin complex other than computer models (12–15). Of particular importance is establishing whether any conformational change in protein C involves the site of activation around Arg^169^, which is thought to become exposed upon binding to the thrombin-thrombomodulin complex (16). Furthermore, it is of interest to establish if protein C has intrinsic conformational plasticity as recently observed in prothrombin, where an equilibrium between open and closed forms (17, 18) directs activation to thrombin along two distinct pathways (19–21). The closed form features an intramolecular collapse of kringle-1 onto the protease domain and promotes activation along the meizothrombin pathway (cleavage at Arg^320^), whereas in the open form the collapse is removed and activation proceeds along the alternative prethrombin-2 pathway (cleavage at Arg^271^).

In this study, we use single molecule FRET (smFRET) and small angle X-ray scattering (SAXS) to probe the structural architecture of the zymogen protein C and the protease APC. We find that both proteins assume a nearly identical conformation in solution, with the constitutive domains vertically stacked in a linear arrangement that does not change significantly upon binding of thrombin.

## Results

### smFRET and SAXS studies

**Figure 2.**
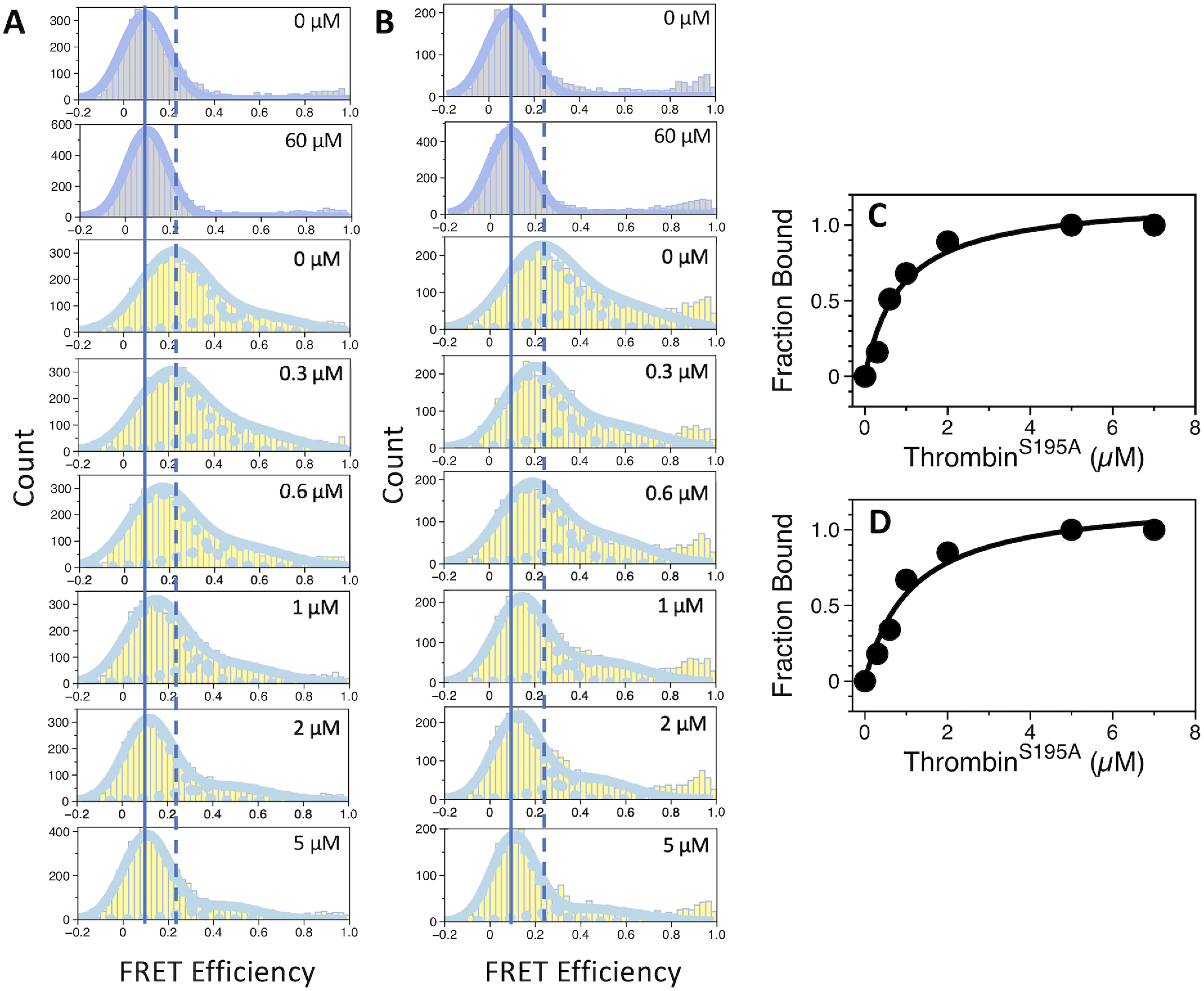
**smFRET profiles of protein C and APC free and bound to thrombin.** Shown are the FRET efficiency histograms of (*A*) protein C and (*B*) APC. Histograms measured in the presence of 5 mm CaCl_2_ are displayed in *gray*, whereas those measured in the presence of 10 mm EDTA are in *yellow*. The concentration of thrombin is indicated. Continuous and discontinuous *vertical lines* mark the center of the FRET populations in the absence of thrombin in the presence of CaCl_2_ or EDTA, respectively. Titrations of protein C (*C*) and APC (*D*) with thrombin were measured in the presence of 10 mm EDTA. Binding isotherms were constructed by following the change in the peak center of the low FRET population as a function of thrombin concentration. Fit of the data to a single-site model yields values of *K_d_* equal to 0.86 ± 0.2 μm (protein C) and 1.1 ± 0.2 μm (APC). Experimental conditions are: 20 mm Tris, 145 mm NaCl, 0.02% Tween 20, pH 7.5, with either 5 mm CaCl_2_ or 10 mm EDTA at 20 °C.

**Figure 3.**
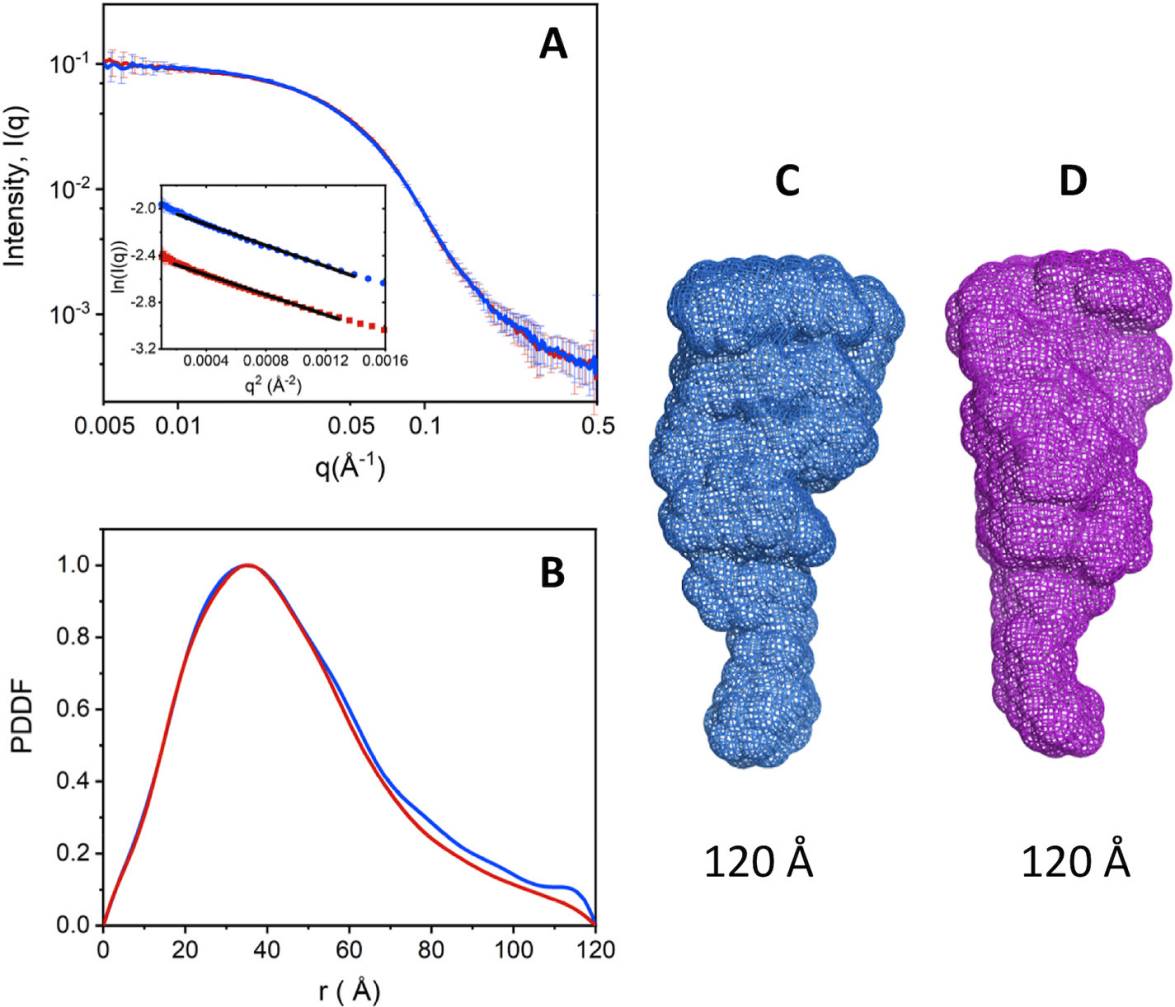
**SAXS measurements of protein C and APC.** *A,* overlaid SAXS profiles for protein C (*red*) and APC (*blue*) superimpose well, indicating that the two molecules adopt very similar conformations. The good linearity of the Guinier plots in the *inset* indicates good monodispersity of the samples. Guinier plots are coded in the same color as their respective SAXS profiles, and the *black solid lines* represent the fit. *B,* PDDF were calculated from the SAXS data in *A*. The value of *D*_max_ estimated from PDDF is about 120 Å for both molecules, which is also supported by 3D SAXS envelope models for APC (*C*) and protein C (*D*).

smFRET measurements with protein C and APC labeled with the AF555/AF647 FRET pair at positions C12/C312 across the Gla and protease domains (Fig. 1*A*) were carried out to evaluate the overall conformational properties of the two proteins. Labeling had no adverse effect on the thrombin-catalyzed activation of protein C, indicating lack of structural perturbations affecting function (Fig. 1*B*). No perturbation was also detected for APC as established from the catalytic activity of the labeled protein compared with the unlabeled protein (Fig. 1*C*). smFRET measurements reveal an interprobe distance between the FRET pair that does not change between the zymogen protein C and protease APC. FRET efficiency histograms are consistent with a single population of labeled species with low FRET efficiency and an interprobe distance of 76 Å (Fig. 2, *A* and *B*). A very small population of labeled species at high FRET efficiency (*E* > 0.9) is also notable in the histogram of APC, but due to experimental uncertainties this population was not considered in subsequent analysis. Our results indicate that the overall architecture of protein C does not change significantly during conversion to APC, thereby establishing another significant difference with the behavior recently reported for prothrombin where activation to meizothrombin is linked to transition from a dominant closed form to the elongated open form (17–19). The conclusion is supported by SAXS measurements that reveal envelopes of protein C and APC with identical maximal particle size (*D*_max_) values of 120 Å (Fig. 3, *A*–*C*).

**Figure 4.**
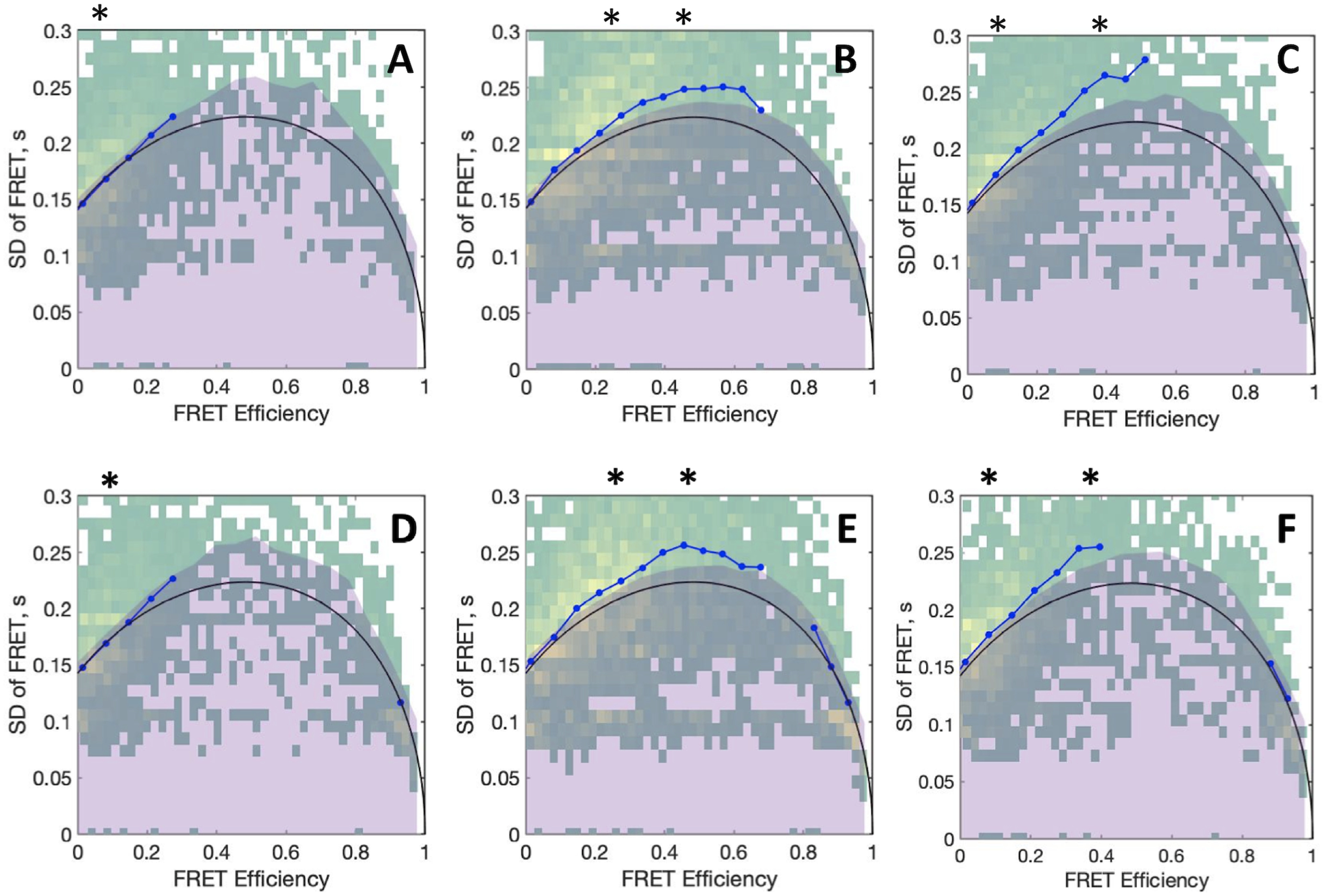
**BVA plots of protein C (*upper panels*) and APC (*lower panels*) measured in the presence of 5 mm CaCl_2_ (*A*, *D*) or 10 mm EDTA without (*B*, *E*), and with 5 μm thrombin (*C*, *F*).** *Asterisks above* each panel denote the FRET population centers obtained from the respective Gaussian fits shown in Fig. 2. In each panel, the expected standard deviation from shot noise is shown as a *black line*, with its 99.9% confidence interval appearing as a *shaded purple* area, whereas the observed standard deviation is shown as a *blue dotted line*. Experimental conditions are: 20 mm Tris, 145 mm NaCl, 0.02% Tween 20, pH 7.5, with either 5 mm CaCl_2_ or 10 mm EDTA at 20 °C.

The linear arrangement of the domains of protein C and APC under physiological conditions is strongly dependent on the presence of Ca^2+^. Chelation of the divalent metal with EDTA produces significant broadening of the FRET histograms and a shift toward higher efficiency (Fig. 2, *A* and *B*). The EDTA-derived histograms are best interpreted with a double Gaussian distribution, yielding peaks with efficiencies of 0.21 and 0.5. Because broadening of the histograms often results from dynamics on the millisecond time scale that occur during diffusion of the molecules through the confocal volume, we performed burst variance analysis (BVA) to establish whether EDTA has an effect on the conformational flexibility of protein C and APC. BVA tests for dynamics in the millisecond time scale by comparing the expected shot noise-limited standard deviation for a given mean efficiency and the experimentally observed standard deviation (22). When molecules experience significant dynamic fluctuations as they transit through the confocal volume, their FRET efficiencies are characterized by an increased standard deviation from that predicted by short noise only (22). BVA plots for protein C and APC are shown in Fig. 4. There are no significant conformational rearrangements on the millisecond time scale when Ca^2+^ is present and the main population with FRET efficiency of about 0.1 displays observed standard deviation that closely matches the predicted one (Fig. 4, *A* and *D*). In contrast, variations above the predicted standard deviation are noted in the presence of EDTA, especially for molecules with FRET efficiencies in the 0.22-0.6 range (Fig. 4, *B* and *E*). Chelation of Ca^2+^ increases the conformational flexibility of the Gla domain, resulting in conformational transitions in the millisencond time scale. Because protein C undergoes conformational changes as it transits through the confocal volume in the presence of EDTA, the values of 0.21 and 0.5 calculated from the double Gaussian distribution represent apparent rather than true transfer efficiencies (Fig. 2).

The observation that Ca^2+^ stabilizes the conformation of the Gla domain is in agreement with results reported by others (23–26). The Gla domain contains multiple binding sites for Ca^2+^ (27–29) and removal of the cation is known to cause significant changes in structural stability (23). Specifically, the far-UV CD spectrum of the isolated Gla domain of protein C is characterized by a significant loss of helical content and increased percentage of random coil elements in the absence of Ca^2+^ (23). The smFRET data suggest that binding of Ca^2+^ to the Gla domain of protein C contributes to the structural integrity of this domain and stabilizes the overall linear arrangement of the entire protein.

smFRET measurements were also used to monitor changes in the conformational properties of protein C during its interaction with thrombin, used as the catalytically inactive mutant S195A to prevent hydrolysis. Binding of thrombin to protein C was confirmed by independent measurements using fluorescence correlation spectroscopy (data not shown). When measurements are carried out in the presence of Ca^2+^, the low FRET distribution of protein C remained largely unaffected (Fig. 2*A*), suggesting that the overall conformation of protein C is already optimized for binding to thrombin and possibly to the thrombin-thrombomodulin complex. Binding of thrombin in the absence of Ca^2+^ causes protein C to assume a conformation similar to the one in the presence of cation (Fig. 2*A*). The same effect is observed when thrombin binds to APC (Fig. 2*B*). BVA plots reveal that the thrombin-bound conformations of protein C and APC, characterized by FRET efficiencies of ∼0.1, display observed standard deviations that map within the upper-limit of the confidence interval of the expected standard deviation (Fig. 4, *C* and *F*). We conclude that thrombin dampens the conformational fluctuations of protein C observed in the presence of EDTA. However, a small fraction of molecules with FRET efficiencies >0.2 may still undergo conformational fluctuations as they diffuse through the confocal volume (Fig. 4, *C* and *F*).

Titration of the shift in the FRET distribution measured in the presence of EDTA allows for quantitative measurements of the interaction with thrombin and yields comparable *K_d_* values of 0.9 ± 0.2 μm for protein C and 1.1 ± 0.2 μm for APC (Fig. 2, *C* and *D*). Thrombin does not show significant binding preference for the zymogen over the protease. The result is in agreement with previous findings (24, 30) and proves that the activation peptide present in protein C but not APC contributes little to the binding interaction with thrombin at equilibrium. The role of the activation peptide is to control the interaction kinetically (31) by hosting residues that decrease the rate of association between protein C and thrombin, especially in the absence of thrombomodulin (16, 25, 32, 33). Much of this effect is due to caging of Arg^169^ by the acidic residues in the activation peptide (16).

### Probing the accessibility of aromatic groups and hydrophobic clusters in protein C and APC

**Figure 5.**
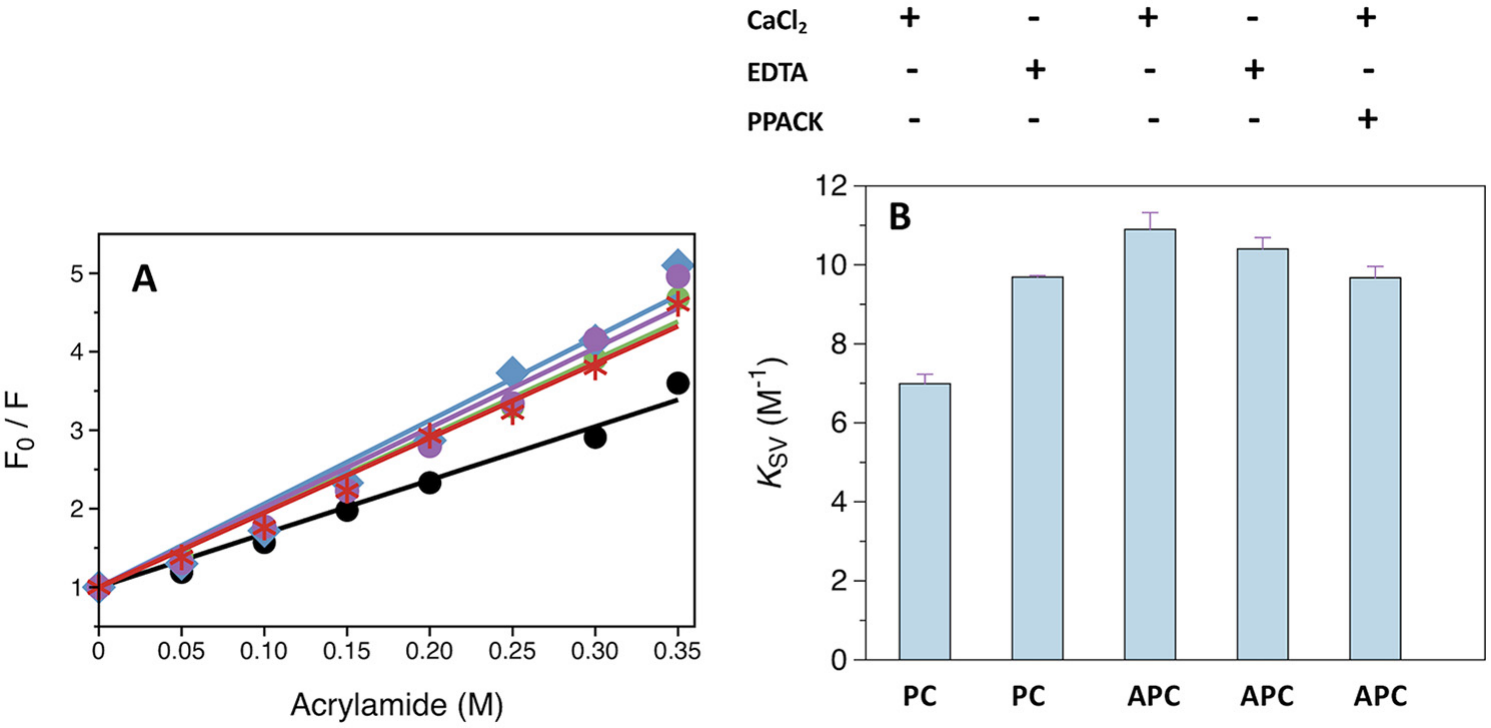
**Acrylamide quenching of the intrinsic protein fluorescence of protein C and APC.** *A,* Stern-Volmer plots measured in the presence of 5 mm CaCl_2_ for protein C (*black*), APC (*blue*), and APC-PPACK (*red*), or in the presence of 5 mm EDTA for protein C (*green*) and APC (*purple*). *B,* graphical representation of the *K*_sv_ values obtained from fitting the acrylamide quenching data to the Stern-Volmer equation (Equation 1). *Error bars* are the standard deviation calculated from two independent measurements. Experimental conditions are: 20 mm Tris, 145 mm NaCl, 0.1% PEG8000, pH 7.5, with either 5 mm CaCl_2_ or 5 mm EDTA at 20 °C.

Acrylamide quenching studies were performed to establish if accessibility of aromatic groups in protein C changes during activation to APC. A graphical representation of the dynamic quenching constant (Equation 1) obtained from analysis of the Stern-Volmer plot is shown in Fig. 5, *A* and *B*. The *K*_SV_ value for protein C is significantly lower than that of APC in the presence of Ca^2+^, showing that activation is linked to increased accessibility of aromatic groups. The higher *K*_SV_ value is not due to a more accessible active site in APC because it does not change in the presence of the irreversible active site inhibitor PPACK. A significantly higher value of *K*_SV_ is also observed in the presence of EDTA for protein C but not APC, suggesting that Ca^2+^ restricts the accessibility of aromatic residues to acrylamide in the zymogen but not the protease. The effect may be due at least in part to the presence of the activation peptide, whose conformation is known to be influenced by the binding of Ca^2+^ (34).

**Figure 6.**
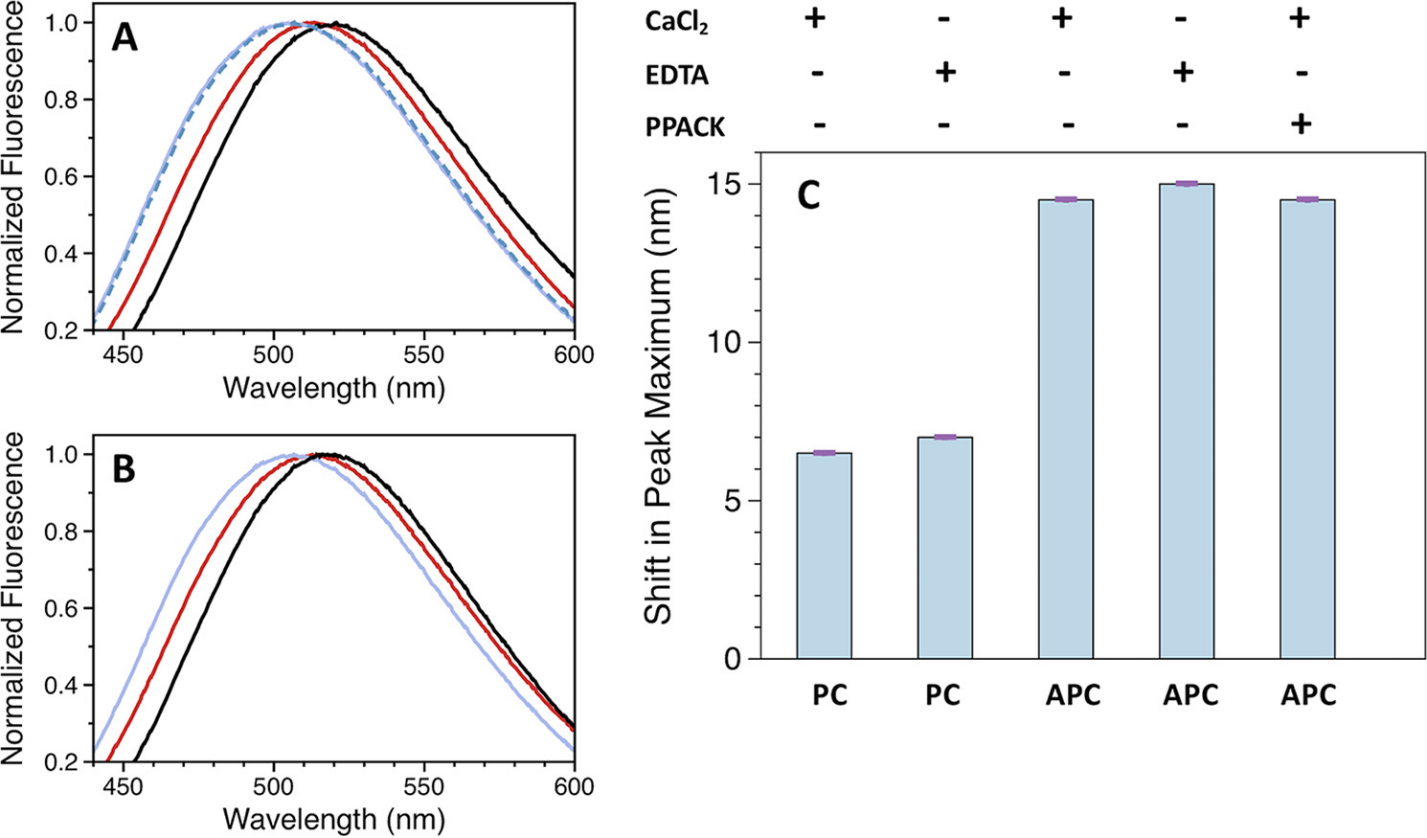
**Reaction between ANS and protein C or APC.** Emission spectra for the reaction with ANS measured in the presence of 5 mm CaCl_2_ (*A*) or 5 mm EDTA (*B*). Colors designate: ANS in the absence (*black*) or presence of protein C (*red*), APC (*purple*), and APC-PPACK (*blue dashed*). *C,* graphical representation of the shift in the emission maximum of ANS measured in the presence of protein C or APC. The difference was calculated by subtracting the maxima obtained for free and protein bound ANS. *Error bars* are the standard deviation calculated from two independent measurements. Experimental conditions are: 20 mm Tris, 145 mm NaCl, 0.1% PEG8000, pH 7.5, with either 5 mm CaCl_2_ or 5 mm EDTA at 20 °C.

Differences in the level of exposed hydrophobic clusters between protein C and APC were detected by monitoring the emission maximum of the fluorescent probe ANS, which becomes progressively shifted to lower wavelengths upon complexing to solvent-accessible hydrophobic groups (35). Upon excitation at 375 nm, the emission spectrum of free 8-anilino-1-naphtalenesulfonic acid (ANS) is characterized by a maximum at 520 nm (Fig. 6, *A* and *C*). In the presence of protein C, the maximum of ANS is blue-shifted to 513 nm, resulting in a difference of 7 nm relative to the free probe. A more pronounced blue shift of about 15 nm is observed when ANS reacts with APC (Fig. 6, *A* and *C*), showing that the level of solvent-accessible hydrophobic clusters increases when the zymogen converts to the mature protease. Again, the increased reactivity of APC with ANS binding to the active site is ruled out by measurements in the presence of PPACK. The level of exposed hydrophobic clusters does not change in the presence of EDTA, for both protein C and APC (Fig. 6, *B* and *C*).

## Discussion

A combination of smFRET and SAXS measurements shows that activation of protein C is not linked to significant changes in the overall structural architecture of the protein. Protein C and APC feature nearly identical SAXS envelopes (*D*_max_ ∼ 120 Å) and interprobe distances for the C12/C312 FRET pair (76 Å) across the Gla and protease domains. The relative arrangement of the auxiliary Gla and EGF domains does not change during the conversion of protein C to APC, in contrast to the conversion of prothrombin to meizothrombin that is accompanied by a drastic relocation of kringle-1, which removes the intramolecular interaction with the protease domain (17–19). Protein C and prothrombin share a common modular assembly, but the N-terminal Gla domain and C-terminal protease domain are connected by two kringles in prothrombin and two EGF domains in protein C and factors VII, IX, and X (36–38). Our results suggest that the presence of intervening EGF domains renders the structure of the zymogen more rigid and similar to that of the active protease. We also note that plasminogen contains kringle domains in its modular assembly and shares with prothrombin a more flexible, intramolecular collapsed architecture that opens up upon activation (39).

Subtle changes between protein C and APC emerge from analysis of the solvent accessibility of aromatic groups and hydrophobic clusters that are more exposed in the protease. The difference may be due to the presence of the activation peptide in the zymogen, but a definite validation will require solution of the crystal structure of protein C. It is also possible that the structure of APC is inherently more dynamic than that of protein C, especially in the <millisecond time scale, thereby allowing more rapid transient exposure of side chains to the solvent.

The results from smFRET studies merit attention because they provide clues on the position of the Gla domain relative to the protease domain. Information on the architecture of the Gla domain of protein C is currently lacking as no structure of the zymogen is available and the only deposited structure of APC refers to a construct devoid of Gla domain (11). Our data indicate a distance of 76 Å for the C12/C312 FRET pair, which is in good agreement with previous ensemble FRET measurements that estimated a distance of 94 Å between the active site of APC and a membrane surface to which the Gla domain was bound (40). The crystal structures of factors VIIa (29) and IXa (41) reveal distances of 86 and 71 Å between the analogous residues that were modified for smFRET measurements in our study. Furthermore, our results reveal that protein C and APC assume an elongated conformation in solution, with comparable *D*_max_ values of 120 Å calculated from SAXS measurements. This conclusion is in agreement with computational models of the zymogen and protease whose vertical axis was estimated to be 130-140 Å long (14). We conclude that vitamin K-dependent coagulation factors carrying EGF domains (protein C, factors VII, IX, and X) assume a conformation where all constitutive domains are vertically stacked. The EGF domains function as static spacers that position the protease domain at a certain distance over the membrane surface to which the Gla domain is bound.

The linear assembly of protein C is stabilized by the binding of Ca^2+^ under physiological conditions. In the absence of Ca^2+^, the Gla domain undergoes conformational changes that occur in the millisecond time scale. Binding of thrombin largely restricts the conformational mobility of protein C and favors a conformation very similar to that bound to Ca^2+^. Our results are in agreement with previous CD measurements with the isolated Gla domain of protein C that have documented a significant loss in total helical content and concomitant increase in random coil-like structures upon removal of Ca^2+^ from the solution (23). Different levels of Ca^2+^-dependent conformational rearrangements have been reported for the Gla domain of other vitamin K-dependent proteins (26, 42–45). Recent bioinformatic analyses have also identified that the Gla domain displays significant intrinsic disorder in the Ca^2+^-free form (46). Therefore, the difference seen in smFRET efficiency for protein C in the presence of EDTA may reflect a partial unfolding of the Gla domain. This is relevant new information on the structural properties of protein C and its mature protease APC that bears on all vitamin K-dependent factors of the coagulation cascade.

## Materials and methods

### Reagents

Thrombin WT and the catalytically inactive mutant S195A were prepared as reported elsewhere (47). Mutations were introduced into the human protein C plasmid carrying a C-terminal HPC-4 tag using a QuikChange Lightning site-directed mutagenesis kit (Agilent Technologies). Baby hamster kidney cells were transfected with the plasmids of interest using X-tremeGENE 9 DNA transfection reagent (Roche Applied Science) according to a standard protocol supplied by the manufacturer. After an incubation period of 48 h, stably expressing clones were selected by incubating the transfected cells with 1 mg/ml of Geneticin. Following clonal expansion, cells were transferred into large cell factories and the growth media from these factories, collected over a period of several weeks, following centrifugation and filtration, was loaded onto a resin that was coupled to the HPC-4 antibody and the protein was purified as described for prethrombin-1 (31). After the immunoaffinity chromatography step, the sample was diluted to achieve a final NaCl concentration below 50 mm and the protein was loaded onto a 1-ml Q-Sepharose Fast-Flow (GE Healthcare) column attached through its top to a 1-ml HiTrap heparin column (GE Healthcare) equilibrated with 20 mm Tris, 50 mm NaCl, 10 mm EDTA, pH 7.5. After detaching the heparin column, the protein was eluted from the Q-Sepharose Fast-Flow column using a 0.05-1 m NaCl gradient. Protein C was further purified on a size exclusion Superdex 200 column (GE Healthcare) equilibrated with 20 mm Tris, 145 mm NaCl, pH 7.5.

Protein C (5 μm) was activated with 7 nm thrombin and 50 nm thrombomodulin after overnight incubation at ambient temperature under experimental conditions: 20 mm Tris, 145 mm NaCl, 5 mm CaCl_2_, 10% glycerol, pH 7.5. The sample was diluted to achieve a final NaCl concentration of 50 mm and the protein was loaded onto a 1-ml Q-Sepharose column attached through its top to 1-ml HiTrap SP HP column (GE Healthcare) equilibrated with 20 mm Tris, 50 mm NaCl, pH 7.0. The upper HiTrap SP HP column, to which the thrombin-thrombomodulin complex predominantly binds, was detached and APC was purified from the Q-Sepharose column using a 0.05-1 m NaCl gradient. Successful activation of protein C and purification from the thrombin-thrombomodulin complex was verified by SDS-PAGE and by monitoring activity with a chromogenic substrate.

### smFRET studies

Protein C and APC were labeled at engineered Cys residues in the Gla (S12C) and protease (R312C) domains (Fig. 1*A*) with Alexa Fluor 555-C2-maleamide and Alexa Fluor 647-C2-maleamide (Invitrogen) using a protocol similar to that used for prothrombin (18). Briefly, protein C and APC (16 μm) were incubated for 1 h in the dark with 2.8-fold molar excess DTT (Sigma-Aldrich) in a labeling buffer composed of 20 mm Tris, 350 mm NaCl, pH 7.5. After excess DTT was removed on a Zeba^TM^ spin desalting column (ThermoFisher) equilibrated with labeling buffer, the proteins (12 μm) were incubated with 2.5-fold molar excess Alexa Fluor 555-C2-maleamide and 2.5-fold molar excess Alexa Fluor 647-C2-maleamide for 2 h with gentle shaking and protected from light. Excess label was removed on a size exclusion Superdex 200 column (GE Healthcare) equilibrated with labeling buffer.

Successful incorporation of the probes at the correct positions was verified by limited proteolysis with thrombin. Samples were run on SDS-PAGE under reducing conditions and visualized on a Typhoon fluorescent scanner after excitation at either 550 or 650 nm. As expected, the label was present in both the light chain (containing residue Cys^12^) and heavy chain (containing residue Cys^312^). Labeling at positions Cys^12^/Cys^312^ did not produce significant structural perturbations as assessed by rates of protein C activation by thrombin and APC hydrolysis of a chromogenic substrate comparable with WT (Fig. 1, *B* and *C*).

smFRET measurements of freely diffusing protein C and APC molecules (150 pm) were collected on a confocal microscope MicroTime200 using pulsed interleaved excitation (PIE) under experimental conditions: 20 mm Tris, 145 mm NaCl, 0.02% Tween 20, pH 7.5, with either 5 mm CaCl_2_ or 10 mm EDTA at 20 °C. Raw data were initially processed with the PIE analysis with MATLAB (PAM) software (48) as described (19). After applying the correct γ-factor and the appropriate correction factors for donor leakage and direct acceptor excitation, data were exported and fitted to a Gaussian function using the peak analyzer function in OriginPro 8.1. All measurements were done in triplicate. BVA was carried out with the PAM program (48). Once the correct γ-factor and the appropriate correction factors for donor leakage and direct acceptor excitation were applied, the analysis was performed using a bin number of 20, a confidence interval sampling number of 100, 5 photons per window, and 120 bursts per bin.

### SAXS studies

SAXS data on protein C and APC were collected at the beamline 12-ID-B of the Advanced Photon Source at the Argonne National Laboratory (Argonne, IL, USA) under experimental conditions: 20 mm Tris, 145 mm NaCl, 5 mm CaCl_2_, pH 7.5. Scattered X-rays at 14 keV radiation energy were measured using a Pilatus 2M detector with a sample-to-detector distance of 2 m. A flow cell was used to reduce radiation damage. Thirty images were collected for each sample and buffer blank. The scattering vector $q=4\pi \lambda^{-1}\sin\;\vartheta /2$ is the momentum transfer defined by the scattering angle ϑ and X-ray wavelength λ. The isotropic 2D images were converted to 1D SAXS profiles, *i.e.* intensity *versus q*, followed by averaging and background subtraction using software packages at the beamline. The radius of gyration, *R_g_*, was determined using the Guinier approximation in the low *q* region (*qR_g_* < 1.3) and its linearity served as an initial assessment of data and sample quality. A value of *R_g_* of about 36 Å was obtained for both molecules. Distance distribution functions (PDDF) were calculated from SAXS data using program GNOM (49). PDDF is the Fourier transform of SAXS data and a weighted distance histogram of atom pairs, and provides an estimate of the maximum dimension *D*_max_ for a molecule. The low resolution envelopes were produced using DAMMIF (50) by directly fitting the SAXS profile with *q* up to 0.40 Å^−1^. Twenty models were generated for every calculation and then aligned and averaged using DAMAVER (51). The normalized spatial discrepancy values of calculations for APC and protein C are about 1.3 and 1.4, respectively, indicating good convergence for individual models. The SAXS profiles for APC and protein C are almost identical, indicating that they adopt a very similar conformation. The 3D envelope reconstruction reveals that both molecules are elongated with *D*_max_ ∼ 120 Å. SAXS data were deposited in the SASBDB database (codes SASDJC6 for protein C and SASDJD6 for APC).

### Acrylamide quenching of intrinsic protein fluorescence

The accessibility of aromatic groups in protein C and APC was studied by monitoring spectra resulting from the acrylamide-dependent quenching of the intrinsic protein fluorescence. Reactions were carried out by incubating 120 nm protein with a specific concentration of acrylamide for 10 min at 20 °C in a buffer composed of 20 mm Tris, 145 mm NaCl, 0.1% PEG8000, pH 7.5, supplemented with either 5 mm CaCl_2_ or 5 mm EDTA. A stock solution of 1.5 m acrylamide (Sigma-Aldrich) was prepared in the same buffer. The effect of H-D-Phe-Pro-Arg-CH_2_Cl (PPACK) (Hematologic Technologies) was studied with APC (1 μm) after incubation for 30 min with 100-fold molar excess PPACK and dilution of the enzyme to 120 nm. Control experiments verified that PPACK completely inhibited APC activity under these conditions.

Data were collected on a HORIBA FluoroMax-4 spectrofluorometer at 20 °C by monitoring the emission at 340 nm upon excitation at 280 nm in a cuvette with a 0.3-cm path length. Data were analyzed using the Stern-Volmer equation,

(Eq. 1) $$\frac{F_{0}}{F}{=1+K}_{\text{SV}}\left[\text{Q}\right]$$

where $F_{0}$ is the fluorescence emission in the absence of acrylamide, F the emission at a specific concentration of acrylamide, $K_{SV}$ the dynamic quenching constant, and Q the concentration of acrylamide. All measurements were carried out at least in duplicate.

### Reaction with ANS

Protein C (1 μm) and APC (1 μm) were incubated with 80 μm ANS (Sigma-Aldrich) for 1 h at 20 °C. For measurements carried in the presence of PPACK, APC was incubated for 30 min with a 100-fold molar excess inhibitor prior to titration with ANS. Control reactions were carried out with 80 μm ANS. Fluorescence emission spectra were collected in the 420-650 nm range following excitation at 375 nm in a cuvette with a 0.3-cm path length. All reactions were conducted in duplicates at 20 °C under experimental conditions: 20 mm Tris, 145 mm NaCl, 0.1% PEG8000, pH 7.5, with either 5 mm CaCl_2_ or 5 mm EDTA.

## Data availability

All data described in the manuscript are contained within the manuscript. The amino acid sequence reported in this paper has been submitted to the Small Angle Scattering Biological Data Bank under accession numbers SASDJC6 and SASDJD6.
